# Endometriosis: New Perspective for the Diagnosis of Certain Cytokines in Women and Adolescent Girls, as Well as the Progression of Disease Outgrowth: A Systematic Review

**DOI:** 10.3390/ijerph18094726

**Published:** 2021-04-29

**Authors:** Jakub Toczek, Żaneta Jastrzębska-Stojko, Rafał Stojko, Agnieszka Drosdzol-Cop

**Affiliations:** Department of Gynecology, Obstetrics and Oncological Gynecology, Medical University of Silesia in Katowice, Markiefki 87, 40-211 Katowice, Poland; zak@czkstojko.pl (Ż.J.-S.); rafal@czkstojko.pl (R.S.); cor111@poczta.onet.pl (A.D.-C.)

**Keywords:** biomarkers, cytokines, adolescence, progression, detection, endometriosis, circulation, non-invasive

## Abstract

Endometriosis is a common chronic gynecological disorder that undoubtedly impacts on quality of life, and is one of the more complex and mysterious illnesses of our century, which is associated with the improper growth of endometrial tissue outside of the uterine cavity. This pathologically implanted tissue can be found most frequently in the minor pelvis, but also in the peritoneal cavity, and can affect many organs, leading to chronic pelvic pain syndrome, infertility, and dysmenorrhea. Endometrial tissue is a particularly dynamic tissue that has a direct impact on the progression of the disease, with altered immunity, as well as cytokine storms within the metaplastic endometriotic site, as possible key factors. Currently, diagnosis of this mysterious chronic illness relies on performing a laparoscopic procedure with tissue sampling. One of the most troublesome outcomes of this unintended progression is that we lack any specific, sensitive, non-invasive diagnostic tools. Currently, the vast majority of regime stewardship options rely on anti-contraceptive drugs, or other remedies that suppress the release of estrogen through the gonads—although in most clinical trials, endometriosis is a chronic progressive disorder that depends mostly on the high concentration of estrogen. Moreover, many specific trials have demonstrated that the eutopic endometrial cells in individuals with endometriosis remain much more resistant to the immunological annihilation process caused by certain elements of the immune system. Nevertheless, eutopic endometrial cells have the potential to similarly escalate the expression of aromatase receptors on the surface of the pathological cells, which in the final cascade cause an increase in the concentration of estrogen, as well as other inflammatory proteins that contribute to pathological outgrowth. Data reveal occurrence among first-degree relatives, suggesting that the specific cascade could be related to inherited as well as epigenetic (acquired) mechanisms. In women with the disease, confirmed by laparoscopic procedures, diagnosis of endometriosis can be established also via detection by gene polymorphism in the genes which are responsible for responsible for the detoxification phase of estrogen receptors and other immunomodulator components. A recent publication aims to reveal a new prospect for the non-invasive diagnosis, detection, and estimation of certain biomarkers for much more specific investigation of the disease’s progression.

## 1. Introduction

Endometriosis is a gynecological disorder defined by the presence of endometrial glands and stroma outside the uterine cavity, related to certain coexistent syndromes, such as pelvic pain (dysmenorrhea, non-menstrual pain, and dyspareunia) and subfertility. Endometriosis is usually chronic and progressive in a subset of patients. This disease is a disabling condition that may significantly compromise social relationships, sexuality, and mental health, but, until now, the exact pathomechanism remained mysterious, as did the percentage of patients with a progression of the disease related to specific markers in their blood serum. The prevalence of endometriosis is estimated at 10–15% among women of reproductive age, and in the young adolescent age group this percentage is seemingly the same. In certain individual studies, in young girls with early menarche, the incidence of endometriosis was greater. Susceptibility to endometriosis depends on complex interactions between genetic, immunological, hormonal, and environmental factors, likewise epigenetic changes. In general, pathological outgrowth is a local pelvic inflammation process, which causes altered function of some immune-related cells—presumably cells of a chronic inflammatory phase, such as macrophages and monocytes, which release certain growth factors, leading to the progression of endometrial plaque changes [1,2,3].

As previously mentioned, it appears likely that the inflammatory and immune cell-related response, along with angiogenic factors and certain cytokines, plays a crucial role in the pathogenesis of endometriosis. The most potent pro-inflammatory cytokines that participate in the development of certain endometrial plaque stages are IL1 alpha and beta. These pro-inflammatory molecules are antagonized by IL-1 Ra, which is a soluble receptor antagonist that causes the action of the pro-inflammatory cytokine to subside. Recent studies show that a decreased soluble concentration of IL-1 Ra leads to the development of disease-related dysmenorrhea. The most prevalent cell in the endometrial plaque is lymphocyte Th2, which secretes IL-4, driving the proliferation of endometrial stromal cells. Furthermore, IL-4 targets other cells, whose ability to respond to this particular molecule varies. Very recent studies emphasize the role of IL-10 as an important anti-inflammatory cytokine that plays a pivotal role in erasing unwanted cell transformation, as well as reducing cellular debris in a silent manner [4,5].

## 2. Classical Blood Biomarkers

**Figure 1:**
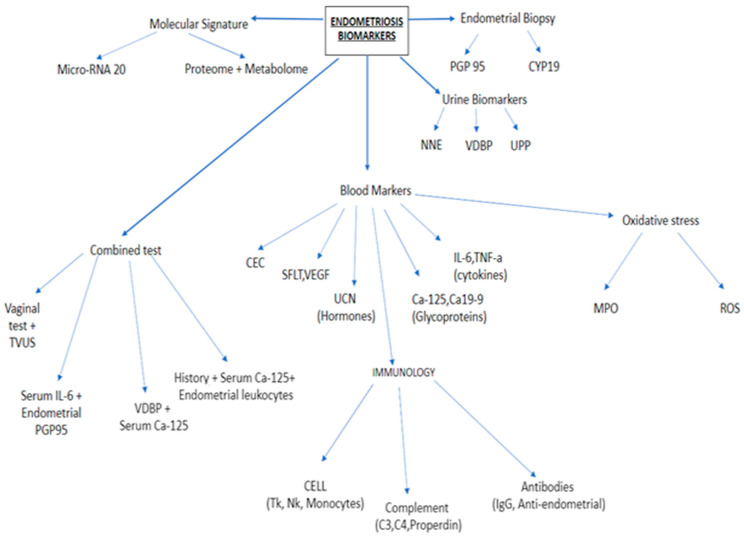
Endometriotic biomarkers [8].

Endometriosis is a complex disorder, which is still not fully comprehendible. Recently, various potential pathomechanisms were considered, such as the regurgitation of menstrual blood, chronic inflammatory milieu, coelomic metastasis, and so forth. This particularly complex disorder comprises of many factors that can participate in the progression of all pathologies, such as hormones, cytokines, angiogenic factors, and other specific glycoproteins. Some of these theoretical constituents could be proposed as endometriosis biomarkers. Figure 1 depicts various differential biomarkers found largely, but not exclusively, in the blood, which have been investigated during the last decade, including apoptotic markers, cell adhesion molecules, and other matrix-related proteins. All of these molecules are involved in a specific, indispensable process during the pathogenesis of endometriosis. Beyond the aforementioned molecules, CA 125, CA 19-9, urocortin, and IL-6 have also been taken as areas of interest as promising, non-invasive biomarkers for early endometriosis. Unfortunately, however, many of these newly revealed markers, which could be taken as indicators of endometriosis, are far from meeting certain criteria for non-invasive diagnosis [6,7,8,9] (Figure 1).

## 3. Role of Inflammation Cytokines and Immunological Molecules as Serum/Plasma Biomarkers

### 3.1. Glycoproteins

There are various candidates within this group, but the most representative molecule is CA 125. Unfortunately, we cannot rely on this single marker, though there are other disorders that can increase the presence of this biomarker during the progression of the disease. A more specific marker in combination with IL-8 is TNF-alpha, which has shown higher specificity during the mid-secretory phase of the menstrual cycle. Another tumor marker, CA 19-9, was investigated in the same way. A recent study indicated that both of these tumor markers were elevated in endometrial samples from affected patients, compared to healthy controls. Unfortunately, the sensitivity and specificity of these molecules were considerably lower than those of CA 125, and it is noteworthy that CA 19-9 correlated much better with the severity of the disease than the usual CA 125 tumor marker. The same applies to glycodelin, which is another molecule responsible for cell proliferation and the neovascular process; glycodelin levels were greatly increased in affected patients, compared to healthy women. The cooperation of these molecules with other factors, such as IL-6, reveals high sensitivity for the detection of endometriosis in the serum probe, or in the peritoneal fluid, during a laparoscopic procedure. The combination of these two independent markers greatly enhances the detection of this chronic gynecological disease. ICAM-1, another glycoprotein, plays a crucial role in the promotion of inflammation and immunological surveillance. Previous studies assess the polymorphism of ICAM-1 along with the progression and severity of endometriosis (there are two polymorphic genes, alleles G/R241 and E/K469). A recent study observed the occurrence of the R241 allele, finding it to be a much more promising prospect in the field of non-invasive detection biomarkers [10,11,12].

### 3.2. Inflammatory Cytokines

In the last decade, many immunological markers especially pro-inflammatory cytokines have been extensively investigated for their non-invasive diagnostic potential. There are several groups representative of certain cytokines, such as IL-1, IL-6, IL-8, TNF-alpha, IFN-gamma, and MCP-1.

(1) IL-6 is one of the pro-inflammatory cytokines found at increased levels in affected women. Most studies estimate that, in stages I–II of the disease, there is an increased level of this particular cytokine, which has a fairly high level of sensitivity, at 76% [13].
(2) IL-8 belongs to the family of chemokines produced and released by cells associated with chronic inflammation—called monocytes/macrophages—and is considered to be a non-invasive biomarker. Many studies have determined that this parameter is drastically augmented during stages I–II of the disease, especially in endometriomas [14].
(3) TNF-alpha is another pro-inflammatory cytokine with extraordinary potential as a pro-angiogenic molecule. Some studies revealed an increased serum level, with the urine level remaining unchanged. The severity of endometriosis greatly depends on the serum level of TNF-alpha. Current research suggests that the soluble TNF-alpha receptor in affected patients shows a potent increase during the follicular phase of the menstrual cycle [15].

### 3.3. Angiogenic Molecules

**Figure 2:**
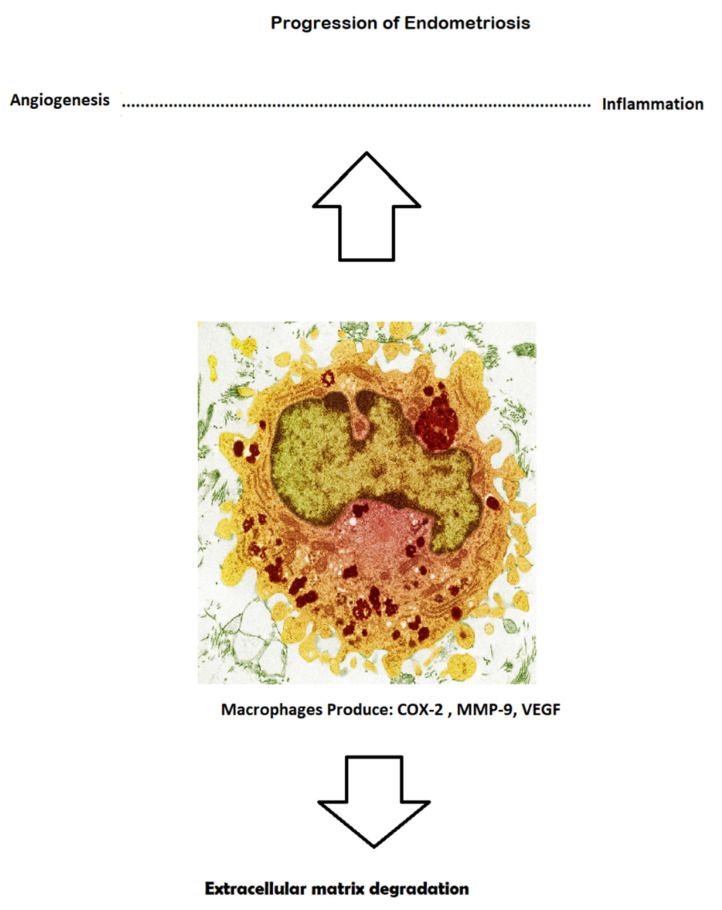
The pathomechanism of endometriosis outgrowth [20].

There are certain angiogenic molecules that have shown encouraging potential to have a significant impact on the pathogenesis of endometriosis: DI-4, angiopoietin, VEGF, and VEGFR; these last two markers possess highly specific features, such as proliferation, migration, and permeability of the cell. Some investigations of IL-17 A as a participant in the angiogenic process have revealed that this molecule emerges as an angiogenic factor, and can upregulate certain molecules, such as VEGF and IL-8. This individual pathological path can induce intraperitoneal angiogenesis in order to preserve the ectopic foci and influence the creation of new outgrowth patches. Furthermore, the surgical removal of eutopic plaques leads to the dramatic decrease of IL-17 A levels in the plasma. VEGF microRNA expression is greater in the hypoxic milieu. The pathological outgrowth of endometrial cells causes hypoxia and induces the production of several molecules with pro-angiogenic potential, such as VEGF, TGF-beta, IL-8, and FGF. As previously mentioned, these factors influence certain features—such as hyper-permeability, the release of plasmatic protein, the formation of fibrin, the proliferation of endometrial cells, and promotion of the process of angiogenesis and fibrinolysis—in the same manner, although many cytokines show pro-apoptotic potential, for instance, IL-8 augments the rate of angiogenesis. IL-4 is responsible for the inhibition process of neo-angiogenesis, which is influenced by FGF, and thus handicaps the transmigration of endothelial cells to FGF. Moreover, IL-1, as part of the pro-inflammatory cytokines group, has shown the features of various augmented angiogenic factors, such as VEGF, IL-8, and FGF. Various studies emphasize that red and white plaques display different intensities of expression for some pro-angiogenic factors. Some studies of endometrial loci during a laparoscopic procedure revealed decreased concentrations of VEGF following danazol treatment, yet simultaneously plasmatic levels of VEGF remained high. PEDF (pigmented epithelial dermal factor) is a factor that mainly inhibits angiogenesis with neurotrophic and anti-inflammatory features and has reportedly been found in endometrial patients. This non-specific marker was found at dramatically elevated levels in plasma, while in excised endometrial ectopic tissue, it was absent. FGF2 (fibroblast growth factor 2) has been recorded in the proposal, both as a feasible biomarker and as a contributor to neovascularization [16,17,18,19,20] (Figure 2).

## 4. New Endometriosis Markers

### 4.1. Urocortin

Urocortin belongs to the family of neuropeptides that are expressed in ectopic endometrial tissue. In recent studies, an increased level of UCN was detected in endometrial plaques, compared to healthy individuals. While the augmented expression of urocortin leads to the development of more extensive symptoms, the depth of invasion similarly enhances the local inflammation process. The location of endometriotic lesions was shown to be associated with UCN transcript levels. Deep infiltrating endometriosis (DIE) was linked with high levels of UCN expressed in ectopic tissue. This non-invasive marker can be used before the operation in order to obtain a predictive value of pelvic pain—either acute, or chronic. In the most investigated group of patients, the levels of UCN-1 were much higher in patients with endometriosis, compared to individuals without specific lesions. Elevated plasma levels were detected in all endometriosis phenotypes (peritoneal plaques, ovarian chocolate cysts, and deep infiltrative outgrowth). The apparent finding of increased UCN-1 plasma levels improves the prospects for diagnosis of endometriosis in symptomatic patients [21,22].

### 4.2. Circulating Endometrial Cells (CECs)

This method is derived from the detection of specific cancer cells in peripheral blood, especially colon and pancreatic cancer. Endometriosis is a benign, pathological, ectopic process that shares some features with malignant cancers—especially dissemination, implantation, and metastasis. The first report concerning the circulation of endometrial cells on the periphery was produced in 2014. These specific markers have greater superiority and specificity than CA 125, but unfortunately, as mentioned previously, most malignant tumors have similar features that lead to the shedding of endothelial cells, which can express the same receptors as endometrial cells—for instance, cytokeratin and estrogen/progesterone receptors, which can interfere with results in the final stages of diagnosis. CECs captured during the mid-secretory phase of the menstrual cycle revealed unique cytomorphological features, including epithelial, stromal, and stem cell-like characteristics. The higher level of circulating cells is closely linked with decidualization of the uterine lining. The CEC hypothesis is based on spread via lymphovascular pathways (also called embolization, metastasis, or transplantation process). Sampson’s vascular spreading theory assumes differences in the anatomical features of normal uterine volume and shape in comparison to a pathological condition; Sampson injected a suspension of endometrial cells into the uterine cavity and discovered that the injected mass of cells escapes from the uterine veins [23].

This theory was further investigated in an experiment in 1940, where Hobbs and Bortnick injected endometrial cell masses into the circulatory systems of rabbits [24]. They found endometrial lesions in the lungs and pleurae of these animals later, during dissection. These cells were later identified via immunohistochemistry. CEC analysis might complete the puzzle of endometriotic pathogenesis. Most likely, cells are shed into peripheral circulation during the decidualization process, where the endometrial cells are chiefly stromal-like, as shown by their gene expression profiles. Epithelial KRT19 cells that were ESR1 positive were usually linked during this phase of the menstrual cycle [25].

## 5. New Molecular Signatures

### 5.1. Proteome

New investigations of proteomic signatures, both in peripheral blood and in the endometrium, have shown promising results. One particular study delineated the detection of six protein peaks (1629.00, 3047.00, 3526.00, 3774.00, 5046.00, and 5068.00 Da) that met the criteria for a SpPin triage test with a sensitivity of 0.66 (95% CI = 0.52–0.77) and a specificity of 0.99 (95% CI = 0.93–1.00). Nevertheless, this proteomic technique has both pros and cons and, unfortunately, is too time-consuming and expensive for regular basis detection [26].

### 5.2. Metabolome

As with all pathological processes in the body, each individual tissue has specific metabolic pathway components—especially ectopic endometrial plaques. Recent investigation of a new metabolic test revealed differences between healthy individuals and females with diagnosed endometriosis. The plasma levels of certain products—such as fucose-, proline-, and choline-containing metabolites—were much higher than normal, and these changes may be linked to the spread and severity of the disease. Some other metabolites—such as lactate, carnitine, B-glucose, and sphingomyelin—were present at abnormal levels in the follicular fluid of ectopic endometrial plaques. Recently, a panel of plasma acylcarnitines was reported as representing a potential diagnostic approach, showing promise as a practical diagnostic tool [27].

### 5.3. MicroRNA

The circulating modulator of gene expression is considered to be a non-invasive marker (attractive candidate), obtainable from pathological endometrial outgrowth. It can also be gathered from other bodily fluids and can be used in the detection of certain pathologies. Microarray technology is a highly sophisticated method that gives us the possibility of investigating systemic levels of miRNA and long non-coding RNA as predictors of endometriosis. Recent data suggest the dysregulation of specific miRNA plays a role in the pathogenesis of endometriosis. Some miRNA induces alterations in ectopic endometrial cells through the persistent modulation of inflammation, proliferation, angiogenesis, and tissue remodeling. In certain studies, Mi-RNA-20a was tremendously dysregulated from the wider family of miRNA molecules, which was associated with endometrial pathological outgrowth [28].

## 6. New Hope as a Biomarker

One group of researchers assumed that miRNA could be a useful marker for shortening the diagnosis time of endometriosis, leading to an early treatment regime and alleviating some symptoms, as well as augmenting patients’ quality of life. Those patients would likely be able to omit unnecessary surgical procedures, which could then open the gate for various therapeutic methods. Among the biggest advantages of this marker is its ability to provide an estimate of the likelihood of recurrence of specific symptoms, allowing for the application of secondary prevention as soon as possible; miRNA is also a time decision marker, because it can be targeted in a specific phase of the menstrual cycle. The novel gene regulator signature can be applied to the detection of pathology in any disease in which altered tissue is a major pathomechanism. This marker can be investigated in urine, serum, plasma, and other bodily fluids. Each miRNA family is a tissue-specific marker. Their systemic decoherence in peripheral circulation points toward distinct pathologies being useful not solely for diagnosis, but also for treatment strategies and for the stratification of the disorder; miRNA has almost every ideal biomarker feature, especially a range for specific disorders, and can be detected early in the disease progression, is non-invasive, is stable, and is related to knowledge of the mechanism. It can be treated differently than a simple marker, due to lower complexity, tissue specificity, and the absence of any known post-translation modification. Many studies note its stability in various bodily fluids, but unfortunately, this biomarker itself is a very short sequence of nucleotides with extremely variable GC content, which possess altered hybridization properties and are difficult to detect. Moreover, the trace amount that is present in serum or urine requires advanced technology to enable a robust, precise, and sensitive technique for miRNA detection. Nevertheless, the use of microarray assays for the detection of a sophisticated biomarker and gene expression modulator in certain bodily fluids is not performed on a regular basis, although most of the specific markers can be detected in young girls to perform diagnosis much faster and less invasively. Regrettably, the database concerning the investigation of miRNA in adolescent girls is lacking [29,30,31].

## 7. Role of MI-RNA and Different Expression in Endometriosis

Most miRNA is of intracellular origin and plays a relevant role in various physiological and pathological processes, including cell-to-cell signaling, cell division, differentiation, and cell death. It is released from cells into peripheral circulation in high-density lipoproteins or packed into extracellular vesicles. Circulating MiRNA in the periphery is degraded by ribonucleases in the blood and other bodily fluids, thus indicating the fragility of Mi-RNA. In the circulation, MiRNA is gathered by reciprocal cells, and is then responsible for the repression of the translation process. A recent study investigated the peripheral circulation microarray profile of MiRNA using a highly sophisticated method and revealed a difference in the expression of MiRNA using qRT-PCR. Surprisingly, many MiRNA families may be involved in the pathogenesis of endometriosis, but in particular, MiRNA-20a was dysregulated, especially in two specific bodily fluids (plasma/serum and peritoneal fluid). These data were confirmed with a group of affected patients. In many other studies, MiRNA is found at different levels in plasma and in serum; usually, a higher level is noted in plasma. Nevertheless, miRNA can be considered a highly specific marker of endometriosis, but there are some technological obstacles related to measuring the primary levels of MiRNA in the blood, as miRNA levels are affected by hemolysis in the circulatory cells. Hence, the error can be seen during measurement, and time sampling collection could possibly affect the primary result concerning MiRNA expression [32,33].

## 8. Factors Impacting miRNA Expression

The specific expression of miRNA in individuals is dynamic, and is influenced by many other non-variable factors, including age, ethnicity, the presence of chronic disease, addiction, and participation in the epigenetic process; miRNA, which is much more specific for endometriosis compared to other chronic diseases, arises from endometrial cells, and is released into peripheral circulation in the form of exosomes. These extracellular vesicles are bound to proteins, and finally modulate them. Many gene modulators are dysregulated or incoherent in tissue and in blood, which influences the process of inflammation. In order to reduce the likelihood of improper bias, all patients who suffer from other inflammatory diseases should be excluded. A crucial determinant of MiRNA the specific phase of the menstrual cycle—it is most likely to be found in the mid-secretory phase, when the dysregulation may be much more emphasized [34,35].

## 9. MiRNA-20a: Crucial Role of Circulating Candidate

**Figure 3:**
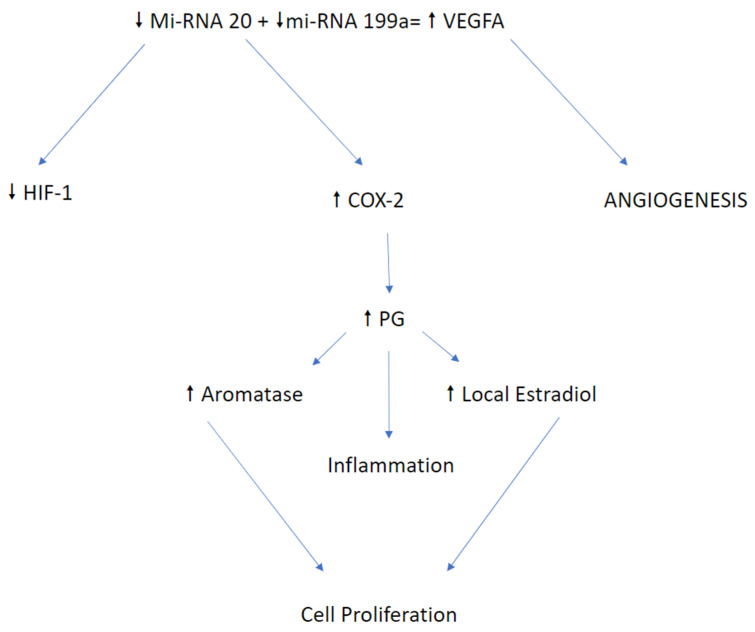
Dysregulation of miRNA-20 (progression cascade) [40].

MiRNA-20a is found in a circulating molecule that was dysregulated and improperly incoherent in more than one study session. This particular gene expression modulator was most frequently dysregulated in the eutopic endometrial tissue. The gene modulator family is very extensive, and miRNA-20a is part of a cluster of specific molecules that are responsible for gene modulation, such as miRNAs-17–92, and as such this molecule plays an essential role in the endometriosis pathogenesis. Hypoxia plays a chief role in the decidualization of endometrial ectopic lesions, though the hypoxia milieu induces the synthesis of prostaglandins, promotes angiogenesis, and has an impact on estrogen receptor modulation. The hypoxic state influences the production of growth factors like HIF-1 and VEGF, and these particular growth factors are among the downstream targets of miRNA-20a. Decreased expression of a particular gene modulator leads to the suppression of the post-translational process—of which RNA molecules are the target, and furthermore influence the production of the growth proteins responsible for distinct functions. HIF-1 stimulates COX-2, which participates in the production of prostaglandins, hence promoting inflammation. Different targets of gene modulators, and other growth factors such as VEGF, promote anomalous and aberrant angiogenesis in endometrial ectopic plaque. For this reason, HIF-1 and VEGF were highly elevated in eutopic endometrial lesions. An additional feature of ectopic endometrial plaques is an augmentation of cell signaling against the reduction of the apoptotic process. The incoherence of miRNA-20a negatively impacts the translation of anti-apoptotic proteins such as BCC2 and CCND1, with E2EF3 as the cell cycle inhibitor. Nevertheless, miRNA-20a targets these two representative factors, and then takes part in epithelial cell proliferation as well as the process of apoptosis. Furthermore, MiRNA-20a also targets TGFB, which has a direct impact on the epithelial mesenchymal transition process, as well as on other pro-inflammatory cytokines, such as IL-8. Dysregulation of miRNA can lead to increases in the release of pro-inflammatory cytokines that provide perfect inflammatory niche and tissue repair, hence affecting the growth of endometrial patches [36,37,38,39] (Figure 3).

## 10. Conclusions

This article is a systematic review that tries to depict a new diagnostic perspective for relevant pathological molecules that participate in the pathogenesis of endometriosis. Many specific molecules can be used for early, non-invasive diagnosis, as well as in the regular follow-ups after the treatment regime. Cytokines and other growth factors are potential biomarkers which may possess great diagnostic potential for the surveillance of this and other chronic gynecological disorders. Undoubtedly, detection of the disease in its early stages can lead to better outcomes and treatment protocols to improve patient quality of life. Unfortunately, the detection of certain biomarkers remains too expensive for regular clinical use. Nevertheless, they are used sporadically among younger patients suffering from endometriosis. Recently, most of these specific markers have shown some potential for the clear diagnosis of the specific stages of endometriosis. They can also be used as markers for observing the progression of the disease. Moreover, the vast majority of patients who undergo laparoscopic procedures currently have no diagnostic tools for such observation. As such, many of these biomarkers can play an essential role in tracking disease progression, in addition to serving as non-invasive diagnostic tools. Our systematic review reveals a significant gap in existing non-invasive diagnostic procedures for adolescent women and young girls, for whom definitive diagnosis using laparoscopic procedures—as opposed to blood serum withdrawal with biomarkers—can pose physical and mental health risks. This may lead to the revelation of new facts about disease progression and the amplitude of biomarker variability, and variation of the levels of certain molecules—along with the stage of disease outgrowth—might indicate a significant role for mention molecules in the pathogenesis of the disease. In order to answer questions concerning the utility of certain non-invasive biomarkers in the early detection of the specific stages, and progression, of the illness, it is necessary to establish the noteworthiness of previous tools for the diagnosis of particular stages of endometriosis, which further establishes the inevitability of the extension of clinical practice to include the use of more non-invasive tools [40,41,42,43].

## Data Availability

All data supporting the reported results can be found in medical journals through PubMed; materials are in accordance with MDPI Research Data Policies.

## Abbreviations

FGF	Fibroblast Growth Factor
VEGF	Vascular Endothelial Growth Factor
PEDF	Pigmented Epithelial Dermal Factor
IL-1	Interleukin 1
IL-1R	Interleukin 1 Receptor
IL-4	Interleukin 4
IL-6	Interleukin 6
IL-8	Interleukin 8
IL-10	Interleukin 10
IL-17	Interleukin 17
TNF-alpha/beta	Tumor Necrosis Factor-alpha/beta
IFN-gamma	Interferon-gamma
UCN-1	Urocortin 1
CEC	Circulating Endometrial Cell
COX-2	Cyclooxygenase 2
TH2	T-Lymphocytes CD40 + helper 2
ICAM-1	Intercellular Adhesion Molecule-1
MCP-1	Monocyte Chemoattractant Protein-1
HIF-1	Hypoxia Induced Factor-1
CA 125	Carbohydrate Antigen 125
CA 19-9	Carbohydrate Antigen 19-9
CCND-1	Cyclin D1
